# A Novel and Highly Effective Natural Vibration Modal Analysis to Predict Nominal Strength of Open Hole Glass Fiber Reinforced Polymer Composites Structure

**DOI:** 10.3390/polym13081251

**Published:** 2021-04-12

**Authors:** Mohammed Y. Abdellah, Mohamed K. Hassan, Ahmed F. Mohamed, Khalil Abdelrazek Khalil

**Affiliations:** 1 Mechanical Engineering Department, College of Engineering and Islamic Architecture, Umm Al-Qura University, Makkah 21955, Saudi Arabia; 2 Mechanical Engineering Department, Faculty of Engineering, South Valley University, Qena 83521, Egypt; 3 Production Engineering & Design Department, Faculty of Engineering, Minia University, Minia 61111, Egypt; 4 Mechanical Engineering Department, Faculty of Engineering, Sohag University, Sohag 82524, Egypt; 5 Mechanical and Nuclear Engineering Department, College of Engineering, University of Sharjah, Sharjah 27272, United Arab Emirates; 6 Mechanical Design and Materials Department, Faculty of Energy Engineering, Aswan University, Aswan 81521, Egypt

**Keywords:** composite laminates, natural frequency, size effect, strength degradation, nominal strength, infrastructure industries

## Abstract

Glass fiber reinforced polymer (GFRP) composite laminates are considered the key material in many industries such as the infrastructure industries and the aerospace sector, and in building structures due to their superior specific strength and lightweight properties. The prediction of specimens’ nominal strength with open holes is still an attractive and questionable field of study. The specimen size effect is referred to its strength degradation due to the presence of holes when specimen geometry gets scaled. The non-destructive test used to measure the nominal strength of such material is a great tool for fast selection purposes, but not secure enough for several purposes. Furthermore, the destructive tests which are more expensive and time-consuming should be avoided in such structures. The present work aims to predict the nominal strength of open-hole GFRP’s composite using modal analysis of their natural frequency as non-destructive tests. At this end, the natural frequency, which is measured using modal analysis procedures, is combined with both linear elastic fracture mechanics (LEFM) and the theory of elasticity to predict the nominal strength of open-hole composite laminates. This advanced model employs two parameters of surface release energy resulting from a simple tension test and Young’s modulus based on vibration modal analysis. It is well established that these types of materials are also subjected to a size effect in dynamic response. Inversely to the known static loading size effect, the size effect in dynamic response increases with specimen size. The novel model gives excellent and acceptable results when compared with experimental and finite element ones. Size effects curves of a nominal strength of these laminates have a very close relative value with those obtained from finite element and analytical modeling. Moreover, the received design tables and graphs would be highly applicable when selecting suitable materials for similar industrial applications.

## 1. Introduction

Glass fiber reinforced polymer (GFRP) composites are widely used in many infrastructure and aerospace industries. Usually, these composites are used in the form of laminates, which have a higher specific strength and lower weight than traditional metals. These types of composites have high corrosion resistance. However, degradation of these composites’ mechanical properties represents a significant constraint to their general use in the petroleum transport pipelines. These composites are considered as quasi-brittle materials, in regard to their fracture behavior. Their behavior in the presence of holes and scaled-up geometries lies in between brittle and ductile behaviors [1]. The change in the (GFRP) composite structures’ mechanical properties because of the size effect produced by the hole involved has been studied in the literature [1]. The impact of inserting some layers of steel mesh is evaluated [1]. Additionally, the attachment improves damage tolerance by increasing ductility while reducing fracture toughness and tensile strength. Moreover, the steel mesh reduces the size effect of the composite plate with an open circular hole.

On the other hand, prediction of nominal strength remains the primary challenge in the design and manufacturing of composite laminates. A rough model for studying the pressure distribution around a circular hole in composite plates was derived [2]. This model was designed using well-known cohesive zone models and is primarily dependent on crack strength and the composite’s fracture toughness. The model attempts to modify and expand the extracted sample size effect curves using two-parameters cohesive laws (linear, exponential, and constant), under biaxial stress. The structural damage plays a vital role to composites used in aviation and space. Effective and reliable structural health monitoring (SHM) systems must provide a probabilistic explanation from the diagnosis [3]. A data-based probabilistic interpretation was obtained to detect damage and performed on the healthy and damaged condition of a honeycomb core sandwich panel such as ANSYS. This proposed method was effective in detecting fissure-type damage in the studied sandwich-type composite structure. It was expected to be suitable for detecting damage in more complex structures [3].

On another study [4], the vibration response of glass fiber and hybrid composite laminates was investigated. The natural frequencies and damping ratio of these two materials were compared with aluminum and tinned steel [4]. They used modal analysis procedures. Based on this study, the researchers concluded that composite structure has a good damping response compared to other monotonic metal. However, the model lacks study of the effect of thickness change and did not give good results for the frequency factor for composite material. The equation of motion for free vibration has been derived by using the third-order shear deformation theory in a composite laminated plate. A sufficient number of plots were introduced for the deformed mode shape in plan and out plan thick laminates at different boundary conditions, where accurate prediction models were obtained for both of the free vibration and mode shape of a thickly laminated transversally isotropic plate [5]. Many works considered the natural frequencies and vibration response of composite material [6,7,8,9,10,11].

Furthermore, the size effect concerning dynamic and vibration for a composite structure used in micro–electro–mechanical-systems (MEMS) devices was investigated [12]. The study was carried out on a copper/laminated glass fiber reinforced epoxy plate with different diameters circular holes and subjected to simple vibration test modal analysis. A simple finite element (FE) model was introduced for obtaining different shape modes; accordingly, the free vibration response of the material developed different trends with holes. With increasing specimen size, the natural frequencies increase, whereas opposite results will be obtained when increasing the static load. A non-grid formula was presented for the analysis of fixed and free vibrations of composite panels using the method of interpolation of radial points of linear modulation [13]. Many examples related to thickness relationships to different spans, material properties, and boundary conditions are considered. The results obtained were compared with accurate solutions and numerical results using other techniques in the literature to verify the performance of the proposed method. Free vibration analysis of a cone plate of functionally gradient material was performed with variable thickness porosity inclusion based on Pasternak foundation [14]. The dynamic response of composite and functional graded material was attractive for many researchers [15,16,17,18,19,20,21]. Due to the importance of modeling methods in analysis and clarification, many of these methods have been proposed, including a fractal model which is also can be used to investigate the mechanical properties of fiber reinforced polymer composite [22,23]. Therefore, and based on the foregoing presented investigations, it appears that there are few studies on size effect analysis in the dynamic response, and also very little numerical investigation to obtain the behavior of composite laminates. Accordingly, this research work presents and addresses three main goals, which lead to a complete understanding of the dynamic behavior of composite laminates. These goals are as follows: (1) the nominal strength of GFRP is predicted using the natural frequency obtained from vibration modal analysis; (2) the size effect in dynamic response will be investigated (3) the important non-dimensional frequency factor (λ) of different modes will be calculated using a finite element method for various hole sizes.

## 2. Materials and Methods

**Figure 1:**
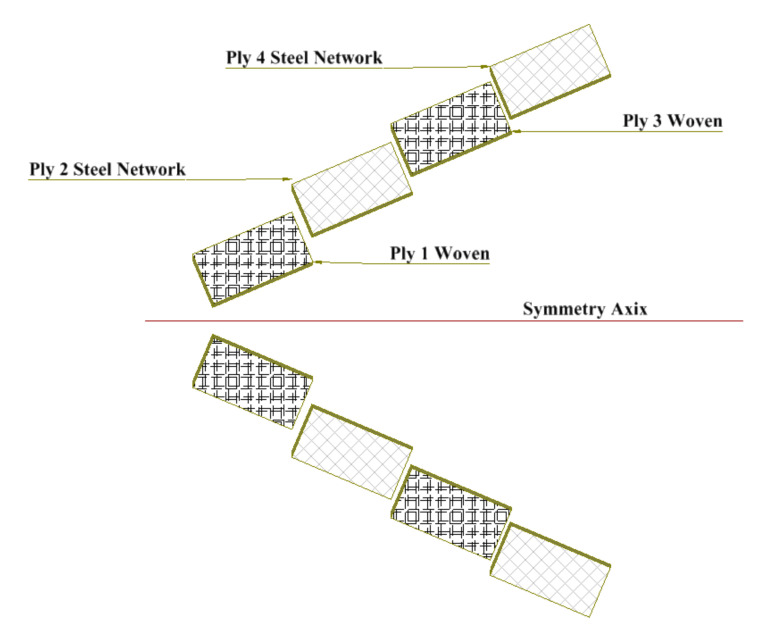
A Schematic for different plies used in manufacturing.

Various complex manufacturing methods were used to make laminated composite structures. Accordingly, the hand-layup method, which is considered the cheapest and simplest, was proposed and selected [24,25]. The manufacturing technique in this method is carried out by procedures where two-sided glass plates are used. One plate is placed as a base and coated by a release agent as wax to avoid sticking. Then after that, a single layer from epoxy material (mechanical properties are listed in Table 1 [26,27]) is uniformly spread over the entire base plate. A glass fiber was placed on the epoxy layer to constitute another layer, followed by repeating the previous step until all the laminate layers were built and completed according to the building sequence as shown in Figure 1. Each laminate contains eight woven glass fiber layers (S1) and a combination of eight layers stuck together four layers from woven glass fibers and four from steel network mats (S2), as shown in Figure 1. The ignition removal technique has been used according to ASTM D3171-99 standard [28]. The mean values were tabulated in Table 2 [4]. The fabricated plates’ thicknesses were average 3.28 mm and 3.08 mm for specimens S1 and S2, respectively.

Six samples with a diameter of 2, 4, 6, 8, 10 and 12 mm were used to test the vibration modal analysis response’s size effect. Still, all these samples confirm that the diameter-to-hole width ratio is constant (d/w = 1/6) [7]. The holed specimens have varying thicknesses; therefore, it is listed in Table 3 for specimens S1 and S2. The mechanical properties of S1 and S2 samples are taken from reference [1].

## 3. Vibration Modal Analysis

**Figure 2:**
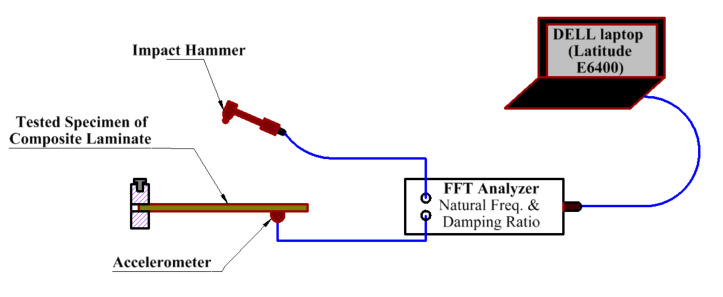
A schematic drawing of measuring system set up.

Modal analysis was performed to the vibration behavior and dynamic characteristics by identifying the damping ratio and natural frequencies. A free vibration test experimentally carries out modal analysis as shown in the experimental layout for modal analysis (see Figure 2). The structure under test consists of various components, with their specifications thoroughly described in References [4,12] and summarized as follows: an accelerometer that measures the vibration levels and sample response at different points on the structure by transducing the vibration motion into electrical signals. The accelerometer was mounted below the specimen by using beeswax (commercial). An impact hammer (Bruel & Kjaer, Nærum, Denmark)with plastic tip used for giving exciting shocks to develop the starting input frequency and magnitude for the modal setup. A six-channel input unit, LAN-XI (Bruel & Kjaer, Nærum, Denmark) used to obtain the sample I/O data. Two input channels were selected for capturing measurements, one channel connected to the accelerometer and the other for the impact hammer. PULSE LabShop (Bruel & Kjaer, Nærum, Denmark) containing 1–6 channel closed licenses were used for data and fast Fourier transform analysis to determine the dynamic properties and model parameters, such as natural frequencies, damping ratios, and mode shapes. These data were extracting from frequency response function curves (FRF).

**Figure 3:**
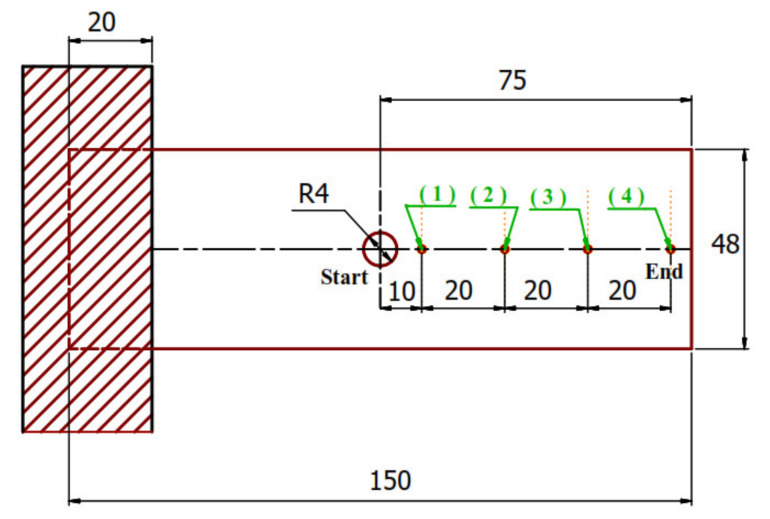
Sample of modal analysis test with holes.

The test specimen with a rectangular cross-section was clamped at one end to a fixture to be considered a cantilever. The sample was firmly fixed to a steel plate and tightened with bolts. Each of the two un-notch specimens (S1 and S2) used as cantilever with dimensions of 150 × 150 mm^2^. All specimen with holes were also used in the same way (see Figure 3).

The LAN XI hardware module captured the vibration data connected to the accelerometer (Bruel & Kjaer, Nærum, Denmark) and the impact hammer through BNC cables and connectors (Bruel & Kjaer, Nærum, Denmark). The hardware unit was connected to a laptop, which had a PULSE LabShop V13.5.0 with a LAN cable (see Figure 2). Each un-notched sample has 20 nodes, whereas open hole samples have 4 nodes, equal distances of 30 mm between them. The mobile shock hammer test technique for each sample was developed to identify the response in all nodes, one at a time, using the program software. The hammer acts as the input exciting for the system by giving hammer impacts at each location for the test specimen. In contrast, system’s response at each node was measured by using the accelerometer. Accordingly, vibration parameters, such as natural frequencies and damping ratios, were obtained by extracting information from the FRF curve at each node in various ways obtained using the program software. Quadratic selection technology is used to determine several positioning shapes. The damping ratios and natural frequencies are assigned at positions 1, 2, 3, and 4. The frequency is obtained for two possible modes [25,29,30,31,32,33,34,35,36].

## 4. Mathematical Modal Analysis

The mathematical solution for vibration concerning a plate with a hole can be identified in the coefficient of damping (*c*) and the coefficient of elasticity (*k*) or flexural stiffness (*D*) [37]. The linear damping force can be measured as:

(1) $$F_{damping}=-\frac{\dot{q}}{Q}$$

where ($Q=\frac{\omega t}{2\pi }$) i is the equality factor and $\dot{q}$ is the generalised velocity the generalized equation of motion for damped simple harmonic motion can be described as:

(2) $$\ddot{q}+\frac{\dot{q}}{Q}+q=0$$

The simple harmonic oscillator Lagrangian with a characteristic frequency.

(3) $$\omega^{2}\equiv -\frac{D}{F}$$

where (*D*) plate stiffness, (F) is the coefficient of the kinetic energy term, and thus *F* > 0 holds. Thus, rescale the time units to define; ($\beta =\sqrt{\left|1/\omega^{2}\right|}=\sqrt{\left|\frac{F}{D}\right|}$) and time co-ordinate (t=βτ).

Such the Q component can be ($Q=\frac{\omega }{b/m}$) and ($\omega =\sqrt{\frac{k}{m}}$) is the natural frequency in rad/sec.

Where x is the amplitude or deviation of the oscillating motion (*m*), ẋ is the velocity (m/s), ẍ is the acceleration (m/s^2^), F is the excitation force (*N*), (ω) is the natural frequency of the mode (rad/sec), and stiffness constant of the plate (*k*) or (*D*) and damping coefficient or damping constant (*c*) of the system. Then it is found that (*Q*) increases as the spring steadies or mass rises relative to the damping constant (*c*). To understand the physical meaning of *Q*, it should rewrite the equations in dimensional form.

The equation of motion of a mechanical vibrated system subjected to a linear damping force is measured as:

(4) $$m\ddot{x}+c\dot{x}+kx=0$$

By when this equation is divided by component (Q), one can obtain the expression for the damping force:

(5) $$F_{damping}=-c\dot{x}=\frac{m\omega }{Q}\dot{x}=\frac{\sqrt{km}}{Q}\dot{x}$$

By divided Equation (5) by component (m), the natural frequency is estimated.

(6) $$\ddot{x}+\frac{\omega }{Q}\dot{x}+\omega^{2}x=0$$

Equation (5) can be rewritten as ($\ddot{mx}+F_{damping}+\omega^{2}mx=0$) for just illustration.

The natural frequency in term of non-dimensional vibration into more familiar units (Hz) can be measured as [38]:

(7) $$\omega_{n}=\frac{\pi }{2}\;\sqrt{\frac{D}{\rho h}}\frac{\lambda^{2}}{l^{2}}$$

where (*h*) is the plate specimen thickness, (l) plate length, (ρ) material density, and D is the cantilever flexural stiffness in the present study:

(8) $$D=\frac{Eh^{3}}{12\left(1-\nu \right)}$$

where *E* is Yong’s modulus and ν is passion young’s modulus, and (λ) is a non-dimensional frequency factor which used for cantilever plate [39]. The problem with open holes changes according to *D* as the open holes specimen has different strengths, which gives different young’s modulus. Thus, the holes’ effect may be simulated using the damping coefficient (*c*) and the plate stiffness *D* [40,41].

### 4.1. Elastic Modulus Perdiction Based on Elasticity Theory

The young modulus E is the more important material characteristic for modal vibration analysis in Equation (8). The young modulus can be rewritten using the rearranged form of this equation as follows.

(9) $$E=\frac{{\left(2\pi \omega_{n}l^{2}\right)}^{2}\rho \left(12\left(1-\nu^{2}\right)\right)}{\lambda^{4}h^{2}}$$

Equation (9) in its form cannot be sensitive to the specimen size; this is because the thickness change only impacts affects it therefore, to insert the effect of specimen size with holes, the following modification would insert in the equation according to [42];

(10) $$E=\frac{{\left(2\pi \omega_{n}l^{2}\right)}^{2}\rho A}{\lambda^{4}I}$$

where ($I=\frac{ah^{3}}{12\left(1-\nu^{2}\right)}$) is the moment of inertia, (λ) is a non-dimensional frequency factor that can be calculated for different modes using Finite element modeling (FEM), and A is the cross-sectional area after subtraction the area of the circular hole measured as in Equation (11).

(11) $$A=ah-\frac{\pi }{4}d^{2}$$

where *d* is hole diameter, a specimen width that specimen width which specimen width changes according to the hole diameter with 1/6 ratio. The non-dimensional frequency factor (λ) is selected by Reference [39] for specimens that have a hole diameter of 4, 6, and 8 mm that are selected from Reference [43]. Similarly, for those with hole diameters 10 and 12 mm, they are chosen using Reference [44].

### 4.2. The Nominal Strength of Open Hole Based on LEFM Theory

The fracture toughness of any cracked specimen according to linear elastic fracture mechanics is based on two parameters un-notch tensile strength ($\sigma_{un}$) and maximum crack opening ($\delta_{c}$) which can be measure by Equation (12) [45] as the following;

(12) $$G_{IC}=\int \sigma_{un}\delta_{c}$$

The critical crack opening can be calculated using classical Equation (13), proposed by Hahn and Rosenfield [46] and Perez [47], under the plane stress condition at fracture:

(13) $$\delta_{C}≅h\varepsilon_{f}$$

where $\left(h\right)$ is the plate thickness, and ($\varepsilon_{f}$) is the fracture strain at which the maximum crack opening can be measured using a simple tension test. Table 4 list the values of calculated crack opening displacement.

Once the fracture strain ($\varepsilon_{f}$) is measured, then hooks law is applied to measure the nominal strength of the open hole specimens of nearly linear behaviors for (S1) material in-plane stress condition as follows.

(14) $$S=\frac{E\varepsilon_{f}}{\left(1-\frac{R}{w}\right)\left(\left(1-\nu^{2}\right)\right)}$$

where $\left(1-\frac{R}{w}\right)$ is notch sensitivity [45]. In case the material flow behaves some plasticity due to steel network inserted between glass fiber layers for specimen S2, the 0.5% strain secant of 2 mm open-hole specimen ($\bar{E}$) is applicable, therefore Equation (15) is modified into the following form. For un-notch specimen hook law is only used without any modification.

(15) $$S=\frac{E\varepsilon_{f}-\bar{E}*0.5\%}{\left(1-\frac{R}{w}\right)\left(\left(1-\nu^{2}\right)\right)}$$

## 5. Finite Element Method

FEM models the vibration behavior analysis. The domain of finite element (FE) model is a simple cantilever beam, with 150 mm length × width mm × thickness (*t*), where the values of thickness and width depend on the open hole tested material specimen (Figure 3). An 8-node linear brick of less integration hourglass control (C3D8R) was assigned for the FE domain. This type of element is characterized by simplicity of use and accuracy in results, that is why it was selected. The fixed end in all directions (encastre mode) for a simple cantilever is the boundary conditions, whereas the other end is free (see Figure 3.). The materials of tested specimens are glass fiber-reinforced epoxy laminates (S1) and glass fiber with steel network reinforced epoxy (S2).

The mechanical properties are listed in Table 5 for material S1 and S2, which are developed as elastic constant and are identified according to the volume fraction of matrix and epoxy using ignition removal method.

The model density depended on specimen geometry and mass; therefore, it is listed in Table 1. At the same time, the mass of each open hole specimen is listed in Table 6 for material S1 and S2.

The model’s governing criteria are Lanczos criteria for eigensolver with acoustic structural coupling, where the frequency is measured at two stable modes. A swept meshing technique is used for meshing the open hole plates because it gives more accuracy. Moreover, an 8-node linear brick, reduced integration, hourglass control (C3D8R) is used for 3-D stress elements. The total number of elements for each hole is (15,660, 16,880, 13,080, 12,300, 14,930, 14,140), (11,680, 16,880, 13,040, 11,990, 12,590, 14,090) respectively for material. element size for all specimen 0.1. The one end is let to be free; the other end is constrained in all directions—the fixation at one end side of the cantilever beam. To select the optimized mesh density and element type; Three refinements (15,660, 13,050, 7830) were chosen; by comparing the obtained frequency value for each mesh density with the experimental results, the closest value was selected and described and by the same manner the optimum number of modes were selected according to the closer average because of comparison. Only the first two modes were measured because the modes that the size or scaling effect is observed. The algorithmic medial type was used with a hex-dominated shape.

## 6. Results and Discussion

### 6.1. Modal Analysis

**Figure 4:**
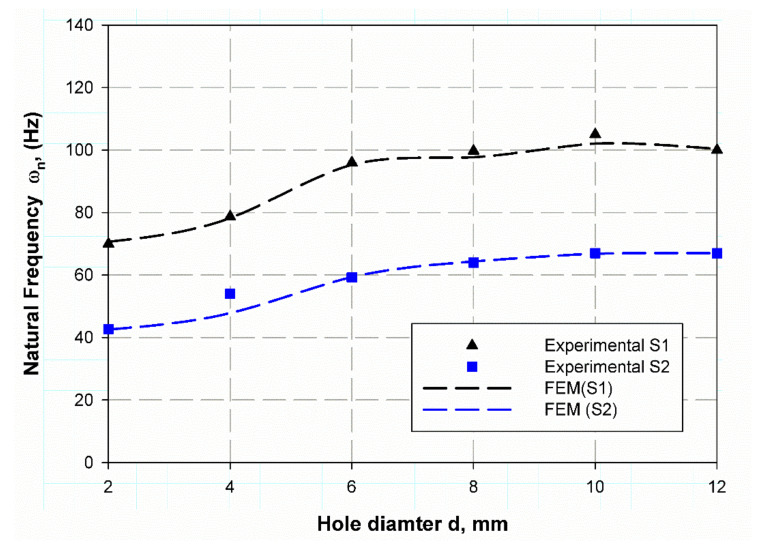
Natural frequency variation with specimen hole at mode 1.

**Figure 5:**
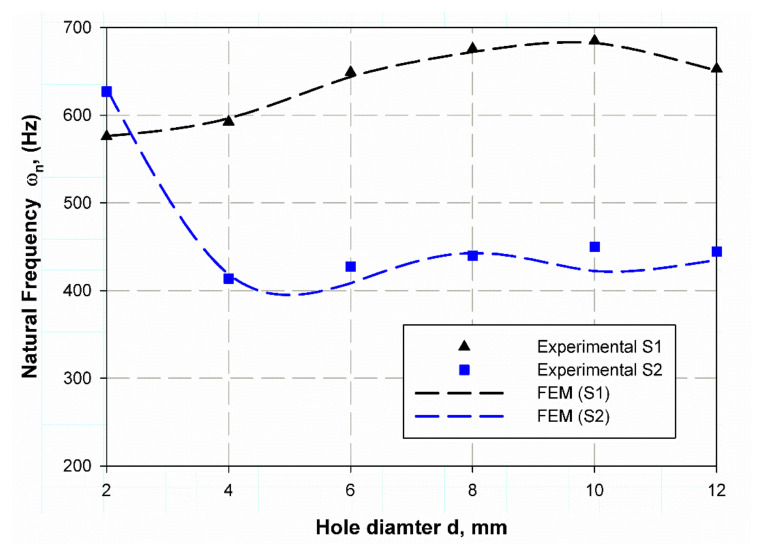
Natural frequency variation with specimen hole at mode 2.

**Figure 6:**
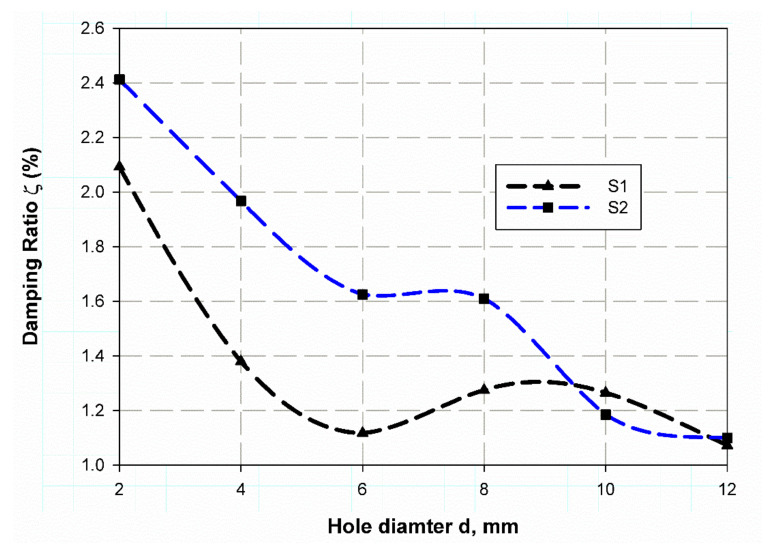
Damping ratio variation with specimen hole at mode 1.

**Figure 7:**
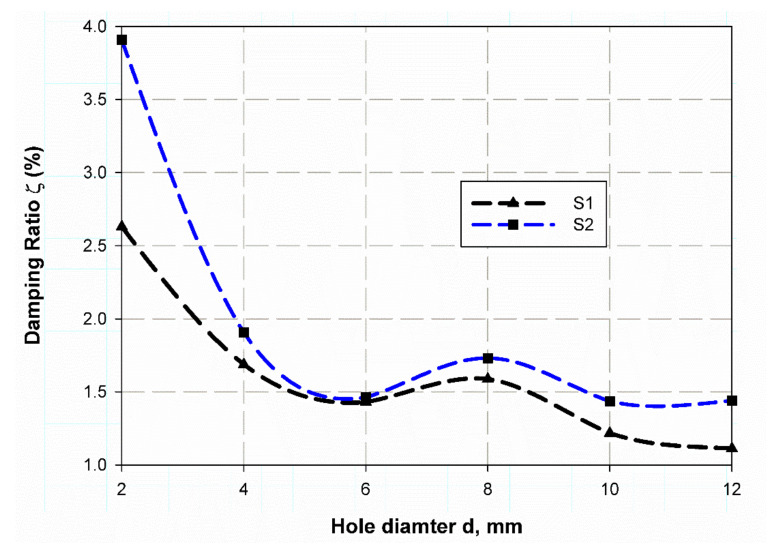
Damping ratio variation with specimen hole at mode 2.

The investigation of experimental modal aims to determine the dynamic behavior, such as natural frequency, damping ratio, and mode shapes of the system configuration during the testing process. Natural frequencies and shapes with which the structure will magnify a load influence can be detected by modal analysis [48]. The first step is to identify the normal frequencies and damping ratios through these analyses, which is carried out by getting information from the frequency response function (FRF) curve. PULSE LabShop automatically supplies the necessary information for both natural frequencies and damping ratios after using different curve fitting. Natural frequency and damping ratios were measured using a modal analysis of the cantilever plate of the un-notch specimen of S1 and S2 material and they are listed in Table 7. It is observed in Figure 4 and Figure 5 that S2 material has a little higher frequency in the first mode (83.1 Hz) while lower frequency (139.8 Hz) in the second mode. In contrast, the first mode has a lower damping ratio (1.9) and higher one (3.25) for the second modes; this is because specimens with steel mesh S2 is heavier than the specimen with glass fiber only S2 (see Table 6); also the steel mesh between glass fiber layers acts as vibration wave dissipation through the rest of material. It is observed that the two first modes have clear values only. Moreover, the frequencies increase with increasing specimen size (see Figure 4 and Figure 5) for modes 1 and 2, respectively. The opposite trend for damping ratios is observed; increasing hole diameter leads to a decrease of damping ratios (see Figure 6 and Figure 7) for modes 1 and 2, respectively. This trend is due to that specimen size increases its cross-sectional area associated with increasing hole radius. The finite element results are in good agreement with the experimental data because finite element analysis takes the real event of the rectangular plate behaviors. The natural frequencies of open-hole specimen on material S1 are clearly shown for mode 1 and 2 (see Figure 4 and Figure 5). The average of four nodes are (70, 78.75, 95.75, 99.75, 105, and 100 Hz) and (575.75, 592.5, 645.25, 675.75, 684.75 and 653 Hz) for (2, 4, 6, 8, 10, 12 mm), respectively. Frequency values rises while the average damping ratio decreases (see Figure 6) in the broad hole diameter range as (2.09, 1.38, 1.12, 1.28, 1.27, 1.07) for Mode 1. Whereas, for Mode 2 (see Figure 7) these values become (2.63, 1.69, 1.43, 1.59, 1.22). The same trend is found for Specimens of material S2 with holes, but with a lower value of natural frequencies (42.75, 54, 59.25, 63.5, 67, 67) and (627, 413.75, 427.25, 439.5, 449.75, 444.25) for mode 1 and 2, respectively. Moreover, the damping ratios are higher than those of S1 material; (2.41, 1.96, 1.63, 1.61, 1.185, 1.1) and (3.91, 1.91, 1.46, 1.73, 1.44, 1.44) for mode 1 and 2 respectively. These trends generally illustrate the size effect on the specimen while in an opposite way, the trend of this influence in response varies about that in the and static loading. In this status, the size effect increases with the specimen size while static loading is decreasing. These results give a similar trend to that of References [12,49], which give a satisfied impression.

It is listed in Table 7 that for material S1, the average natural frequency 78.368 and damping ratio 2.39, and they are 178.315 and 2.4, for mode 1 and 2, respectively. Whereas, for material S2 have a higher natural frequency of 83.1 and a lower damping ratio in the case of mode 1, while it has a lower frequency of 139.8 and a higher damping ratio in the case of modes.

### 6.2. Frequency Factor Prediction Using FEM

**Figure 8:**
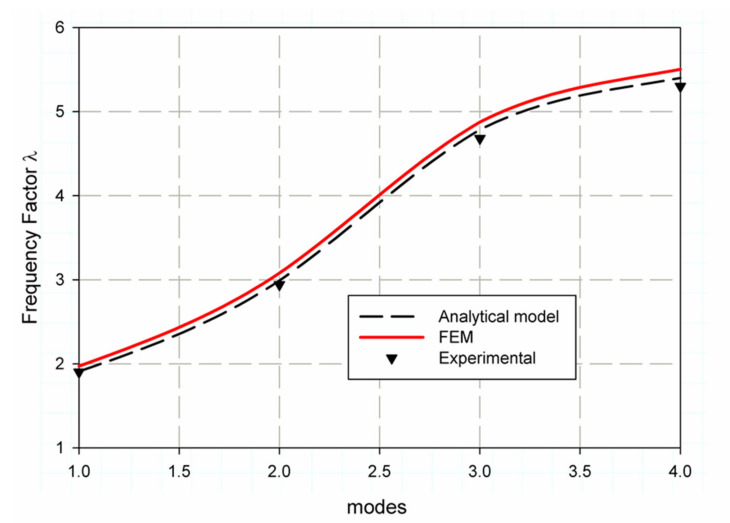
Non-dimensional frequency factor (λ) for S1 material.

**Figure 9:**
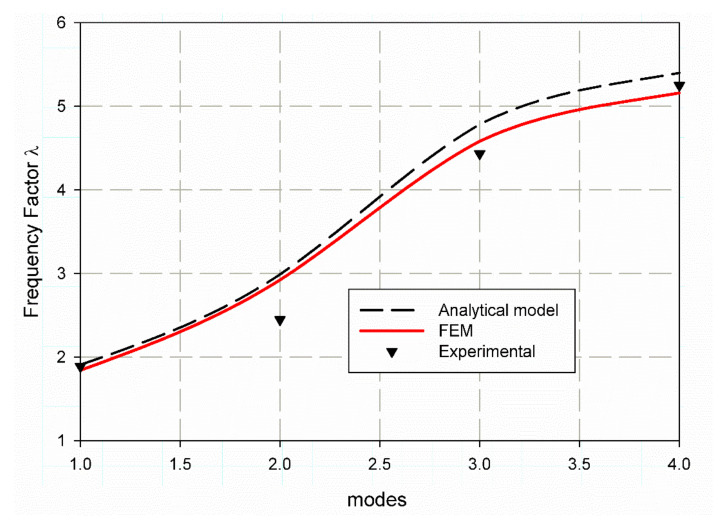
Non-dimensional frequency factor (λ) for S2 material.

To validate the finite element model to be applicable for the prediction of the non-dimensional frequency factor (λ), firstly, the finite element model is used to calculate the non-dimensional frequency factor (λ) for un-notch composite square plate and then compared with that of Reference [43]. It is clearly shown in the data which are listed in Table 8, that the error percent, when compared with the data in Reference [36], decreases for specimens of S1 material with modes increment as (3.3, 2.97, 1.91, 1.92), while for specimens of material S2 the % error increases as (3.44, 2.21, 4.19, 4.42). These results are clearly shown in Figure 8 and Figure 9. This behavior can be attributed to the complex environment of laminated composite material, which has many interactives between internal layers and fiber compared with isotropic plates. In award, these results are considered acceptable with these small values of error from a scientific point of view [50]. The following equation calculates the experimental values. $\lambda =\frac{\omega_{nexp.}}{\omega_{n}}$, where ($\omega_{nexp.}$) is the natural frequency obtained from experimental modal analysis and ($\omega_{n}$) the frequency measured from Equation (8) without λ. It was shown in (Figure 8) that for specimen S1 the non-dimensional frequency factors λ the values are (1.9721, 3.0787, 4.8744, 5.5029) these FE results (red solid lines) are very close to the analytical data (black dash line) [43] and experimental results (black triangles). Similarly, for specimen S2 (see Figure 9), the FE data are (1.8432, 2.9238, 4.5822, 5.1602); these data are very close to experimental results but differ from the increase in analytical data in modes three and four. This is due to that for specimen S2, the steel meshes dissipate waves and change the mass and density of the composite which lead to inaccuracy of analytical solution.

**Figure 10:**
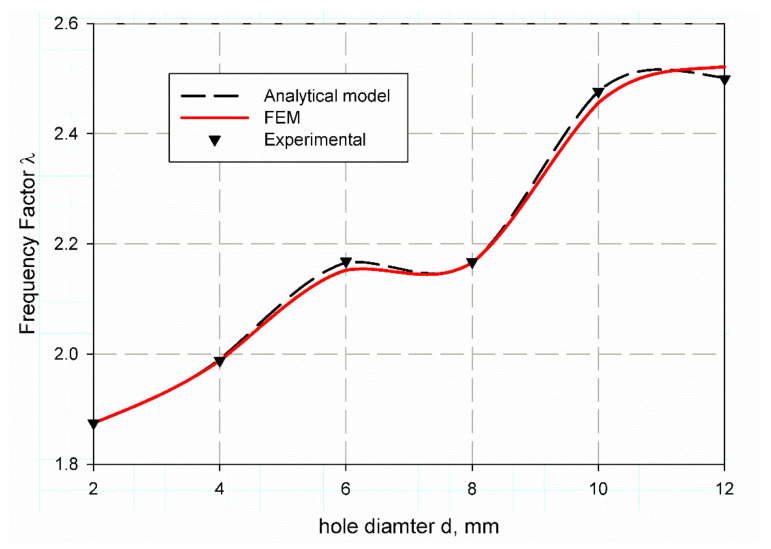
Non-dimensional frequency factor (λ) for open hole specimen of S1 material.

**Figure 11:**
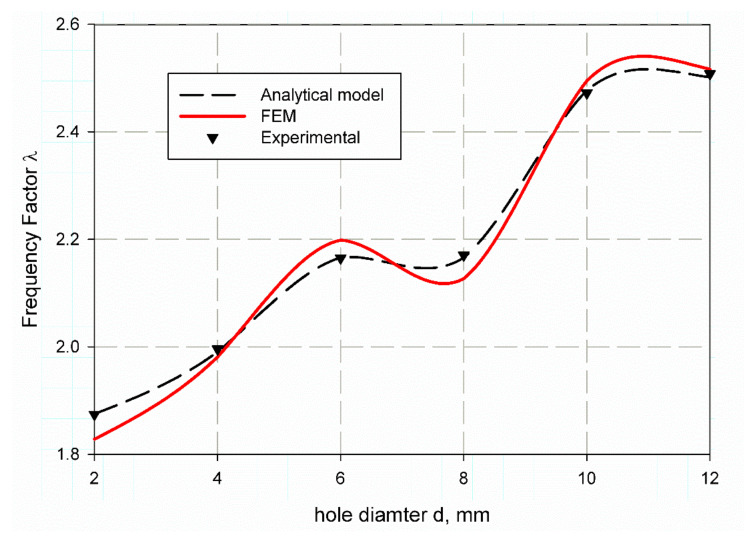
Non-dimensional frequency factor (λ) for open hole specimen of S2 material.

Table 9 tabulated the value of the predicted non-dimensional frequency factor (λ) using finite element analysis for both mode 1 and 2 for material S1. It is observed that with hole increases from (2 mm to 12 mm) the values of non-dimensional frequency factor (λ) are quit increases as (1.8749, 1.9882, 2.1524, 2.1663, 2.4563, 2.5217), and (5.374773, 5.41222, 5.618912, 5.64573, 6.279644, 6.401345) for mode 1 and mode 2 respectively. Nearly, same results for specimens of S2 material that listed in Table 9 as (1.8279, 1.9812, 2.1985, 2.1270, 2.4950, 2.5170), but the value of 2 mm holes has an irregular sense for mode 2 as it is (7.207791) then return to be regular for (4, 6, 8, 10 and 12) mm holes as (5.530115, 5.793477, 5.598823, 6.444072, 6.443426). This may be due to an error in the manufacturing or test fixation of this specimen. For more understanding and observation, Figure 10 and Figure 11 are considered. It is observed that the non-dimensional frequency factor (λ) increases as the hole diameter increase for both specimens S1 and S2 as shown in (Figure 10 and Figure 11). λ value increases sharply until specimen of 6 mm hole diameter, then decreases sharply, afterthought returns to increase with specimen of 10 mm diameter and finally reaches to stability at diameter of 12 mm. S1 and S2 exhibit nearly the same trend but the model gives large error at specimens with 2 mm, 6 mm and 8 mm hole diameters for S2 as i shown in Figure 11.

### 6.3. Strength Prediction

**Figure 12:**
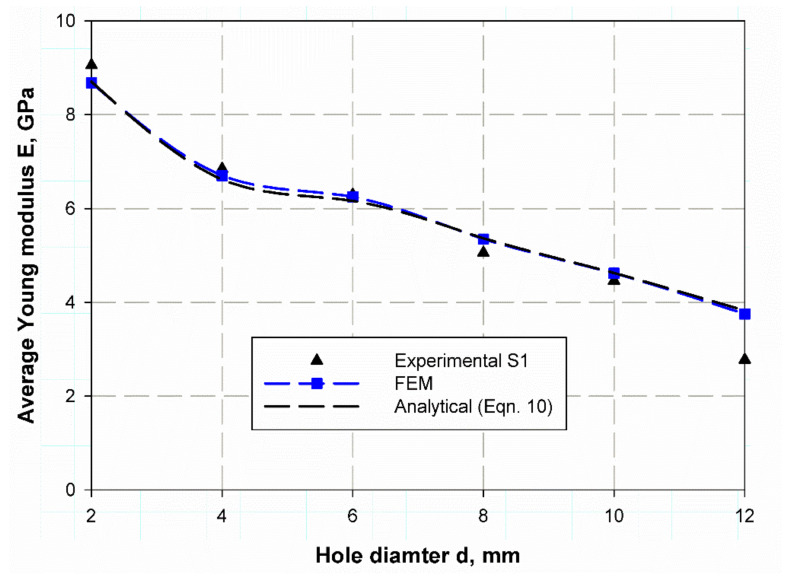
Young modulus prediction of S1 specimen based on reference [43].

**Figure 13:**
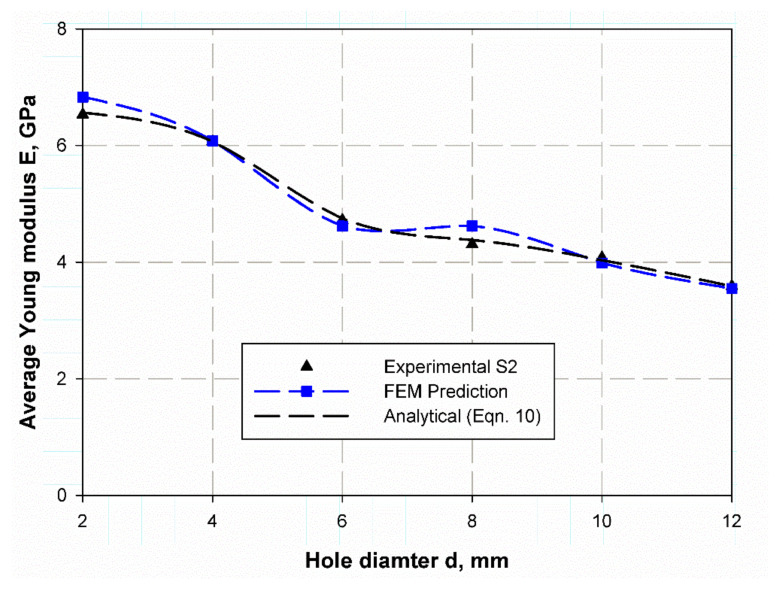
Young modulus prediction of S2 specimen based on reference [43].

At the first step, the Young’s modulus of the open hole specimen should be measured using Equation (10) based on natural frequency and non-dimensional frequency factor (λ) listed in previous tables. Figure 12 and Figure 13 illustrate the predicted Young modules for both S1and S2 specimens where the prediction is based on an analytical solution of Equation (8) and the FE results are based on the FE prediction of non-dimensional frequency factor (λ). The obtained results from the analytical, FE, and experimental data are very close but, there are differences among them. This deference increases in S1 with 12 mm hole due to the complicated behaviors of composite laminates. There are also variances in the predicted values as in specimens of hole diameter (2, 4, 6) mm, the error is negative. In contrast, for the sample of (8, 10, 12) mm hole diameter, it is positive. Upon operation, the positive difference is considered a good and more secure than those of negate one. Therefore, this model may be considered as a non-destructive test that can be used to calculate the equivalent Young modulus which is the most important elastic characteristic and mechanical property of any material especially composite laminates.

**Figure 14:**
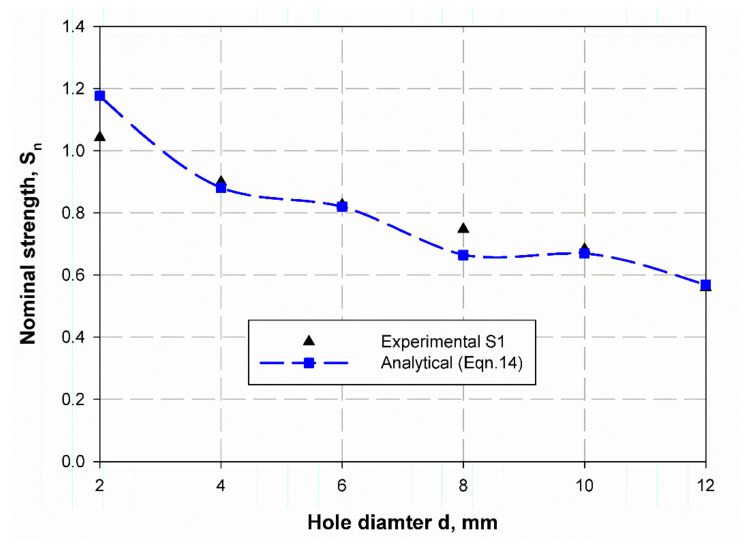
Nominal strength prediction of S1 material using Equation (14).

**Figure 15:**
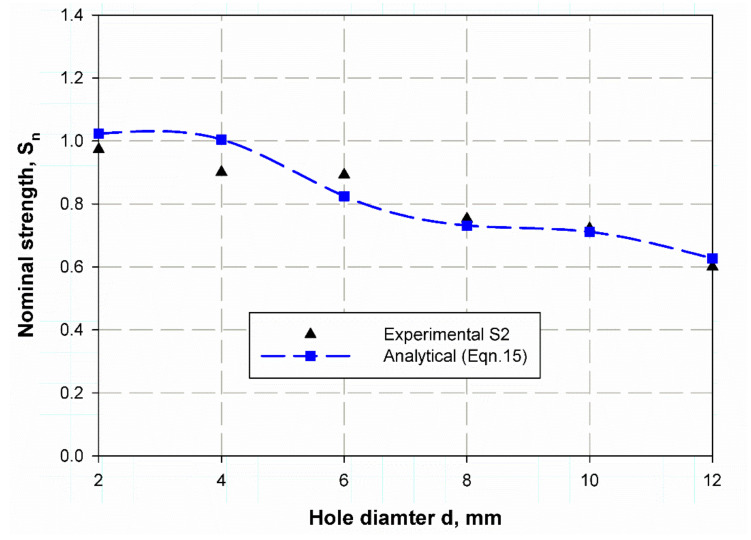
Nominal strength prediction of S2 material using Equation (15).

Table 10 listed a comparison between the predicted nominal strength using the present analytical model Equation (14) and experimental ones for S1 and S2. The predicted nominal strength. For S1, it shows very close values to the experimental data with the error percentage (%) of (0.11, 0.72, and 0.11) for un-notch and specimen with (6 mm and 12 mm) open hole diameters, respectively. Whereas these results have a moderate value (2.06, 1.99) for the specimen with 4 mm, and 10 mm holes diameter. Large values (−12.79, 11.14) for the specimen with 2 mm, and 8 mm holes diameter are observed. The same trend for the specimen of material S2 predicted by Equation (15), listed in Table 10, but the error percentages (%) have different values where the close value (0.63) is gone to specimens with 8 mm open holes diameter, the moderate’s values (−5.79, 6.79, 2.96, −4.44) are for specimens with (2, 6, 10 and 12) mm diameters hole, while the large value (−12.79) is for 2 mm diameter hole. However, for safety, the larger values of the error percentage (%) of both materials S1 and S2 is negative, which means it is more secure. The size effect curve is shown in Figure 14 and Figure 15 for material S1 and S2 respectively, it gives a good prediction for the nominal strength with very close accuracy for a specimen which has hole diameters of 2, 4, 10, and 12 mm in S1 material, while it is getting large (Figure 14) in a specimen with 2- and 8-mm hole diameter. Moreover, in the specimen of material S2 all specimens give a quite relative value except those with hole diameters of 4 and 6 mm. The model has the advantage to measure the size effect of the composite specimen using the natural frequency without destroying it, as in LEFM and cohesive zone models based on destructive tests.

## 7. Conclusions

The size effect in quasi-brittle material such as GRP’s composite laminates is commonly known under static loading as the decrease of nominal strength with specimen size increase with scaled open holes. It is reported that the size effect is present in the case of dynamic response; the present size effect is in the form of natural frequency which increase with increasing of specimens’ hole diameters. This enables the idea that to extract the specimen size effect and hence predict a nominal strength of open holes composite specimen using natural frequency to be a non-destructive test procedure. The obtained results of non-dimensional frequency factors can be considered as design tables for different diameter open holes, composite laminates, and hybrid ones. The finite element model gives good agreement results when compared with experimental and published results. Generally, the model would be a useful tool in material selection purposes for fast determinations of the cracked GRP’s composites strengths. The present model addresses a non-destructive tool to measure the nominal strength of open holes specimen and provides a robust tool with cheapest and economic method which will save the composite material that is considered expensive. Moreover, it enables ease determination of the composite material nominal strength which are used airplanes or any infrastructure industry without need to perform destructive test. The model still needs to be implanted to the parametric study based on the natural frequency of a composite and the specimen dimensions such as thickness, width, length, and hole diameter. Moreover, fractal modeling may be considered in analyzing such type of experimental results, so it is recommended to perform both parametric study and fractal modeling in near future.

## Figures and Tables

**Table 1:** Mechanical and physical properties of E-glass fiber and epoxy resin [26,27].

Properties	E-Glass	Kemapoxy(150RGL)
Density (kg/m3)	2540	107 ± 2
Tensile strength (MPa)	2000	50–100
Tensile modulus (GPa)	76	1.2–4.5
Passion ratio	0.25	0.35
In plane shear modulus	30.8	1.24
Failure strain		1.7

**Table 2:** Characteristic properties of un-notch used material [4].

Properties	S1	S2
Density (kg/m^3^)	1650	1850
Equivalent Young’s modulus (GPa)	13.7793	5.6
Volume friction %	$\nu_{f}=30$	$\nu_{fglass}=25.5,\nu_{fsteel}=1.1$
Passion ratio	0.25	0.25

**Table 3:** Mechanical properties and thickness of specimens S1 and S2.

Diameter (mm)	Thickness (mm)	
	S1	S2
2	3.30	3.50
4	3.35	3.30
6	3.35	3.00
8	3.60	3.00
10	3.08	2.90
12	3.00	2.80
Average	3.28	3.08

**Table 4:** Surface release energy and corresponding crack opening displacement for S1 and S2.

Specimen	G_IC_ (Experimental) KJ/m^2^	Crack Opening, µmm
S1	31.54	7.50
S2	30.39	9.12

**Table 5:** Mechanical properties of glass fiber-reinforced epoxy laminates S1 and S2.

Properties	Value	
	S1	S2
E1 (GPa)	24.6	8.18
E2 (GPa)	24.6	8.18
E3 (GPa)	5.30	5.30
G1, G2, G3 (GPa)	2.18	1.20
µ1, µ2, µ3	0.17	0.17

**Table 6:** Different masses of various holes for S1 and S2 material.

Diameter (mm)	Mass (mm)	
	S1	S2
2	8.09	17.38
4	16.92	30.13
6	25.71	39.46
8	36.76	51.28
10	42.12	69.27
12	53.32	96.20
Average	30.49	50.62

**Table 7:** Natural frequencies and damping ratios of specimen S1and S2 [4].

Node Number	Mode 1				Mode 2			
	Natural Frequency (Hz)		Damping Ratio (%)		Natural Frequency (Hz)		Damping Ratio (%)	
	S1	S2	S1	S2	S1	S2	S1	S2
average	78.37	83.1	2.39	1.90	178.32	139.8	2.40	3.23

**Table 8:** Non-dimensional frequency factor (λ) (FEM) of un-notch S1 and S2.

Modes	S1	%Error	S2	%Error	Reference [43]
1	1.9721	3.3	1.8432	3.44	1.909
2	3.0787	2.97	2.9238	2.21	2.990
3	4.8744	1.91	4.5822	4.19	4.783
4	5.5029	1.92	5.1602	4.42	5.399

**Table 9:** Non-dimensional frequency factor (λ) (FEM) for notch specimens S1 and S2.

Specimen	Mode 1		Mode 2	
	S1	S2	S1	S2
2	1.8749	1.8279	5.374773	7.207791
4	1.9882	1.9812	5.412220	5.530115
6	2.1524	2.1985	5.618912	5.793477
8	2.1663	2.1270	5.645730	5.598823
10	2.4563	2.4950	6.279644	6.444072
12	2.5217	2.5170	6.401345	6.443426

**Table 10:** Nominal Strength prediction of open hole specimens of S1 and S2 material.

Specimen	S1		% Error	S2		% Error
	Strength (Experimental) MPa	Strength (Predicted) MPa		Strength (Experimental) MPa	Strength (Predicted) MPa	
Un-notch	208.76	209	0.11	166.5	163.47	1.82
2	218	245.88	−12.79	162	170.35	−5.79
4	188	184.12	2.06	150	167.24	−11.49
6	172.7	171.46	0.72	148.6	137.30	6.79
8	156.3	138.88	11.14	125.35	121.81	0.63
10	142.8	139.96	1.99	120.26	118.45	2.96
12	117	118.79	0.11	100	163.47	−4.44
